# Fmrp targets or not: long, highly brain-expressed genes tend to be implicated in autism and brain disorders

**DOI:** 10.1186/s13229-015-0008-1

**Published:** 2015-03-11

**Authors:** Rebecca L Ouwenga, Joseph Dougherty

**Affiliations:** Department of Genetics, 4566 Scott Ave, Campus Box 8232, 63110-1093 St. Louis, MO USA; Department of Psychiatry, Washington University School of Medicine, St. Louis, MO USA

**Keywords:** FMRP interactome, Autism, Genome-wide association

## Abstract

**Background:**

Many studies have demonstrated a robust statistical overlap between genes whose transcripts are reported as Fragile X Mental Retardation Protein (Fmrp)-binding targets and genes implicated in various psychiatric disorders, including autism. However, it is not clear how to interpret this overlap as the Fmrp protein itself is not considered to be central to all instances of these conditions.

**Findings:**

We tested whether Fmrp binding may be a proxy for some other features of these transcripts. Reviewing recent literature on the cross-linking and immunoprecipitation (CLIP)-derived targets of Fmrp in the brain, and the literature on identifying genes thought to mediate autism and other psychiatric disorders, reveals that both appear to be disproportionately made up of highly brain-expressed genes. This suggests a parsimonious explanation—that the overlap between Fmrp targets and neuropsychiatric candidate genes might be secondary to simple features such as transcript length and robust expression in the brain. Indeed, reanalyzing Fmrp high-throughput sequencing of RNAs isolated by CLIP (HITS-CLIP) data suggests that approximately 60% of CLIP tag depth can be predicted by gene expression, coding sequence length, and transcript length. Furthermore, there is a statistically significant overlap between autism candidate genes and random samples of long, highly brain-expressed genes, whether they are Fmrp targets or not.

**Conclusions:**

Comparison of known Fmrp-binding targets to candidate gene lists should be informed by both of these features.

**Electronic supplementary material:**

The online version of this article (doi:10.1186/s13229-015-0008-1) contains supplementary material, which is available to authorized users.

## Findings

### Introduction

In 2011, Darnell *et al*. published a study on the Fragile X Mental Retardation Protein (Fmrp) that demonstrated through brute-force biochemistry and elegant informatics, a fundamental role for Fmrp in stalling of ribosomes in the brain [1]. Included was a table of the RNAs identified as bound to Fmrp. While the authors were careful to note their analysis likely “…underestimates the true number of Fmrp-regulated mRNAs,” this table has gradually become taken as the *de facto* Fmrp regulon - the comprehensive set of transcripts regulated by Fmrp. Since then, it has become recurrent in the psychiatric genetics literature to examine the intersection between the risk genes of a disorder and these Fmrp targets, often demonstrating a significant overlap between the two (for example, [2-8]). However, while statistically significant, these results are difficult to interpret. Does this mean the Fmrp protein is central to all of these diseases and processes? Or is Fmrp binding serving as a proxy for some other features of the genes that may parsimoniously explain their contribution to genetic risk? Here, we test a simple alternative explanation for these Fmrp-related findings: both these Fmrp targets and genes that moderate neurocognitive traits contain a disproportionate number of long and highly brain-expressed genes.

## Results

**Figure 1:**
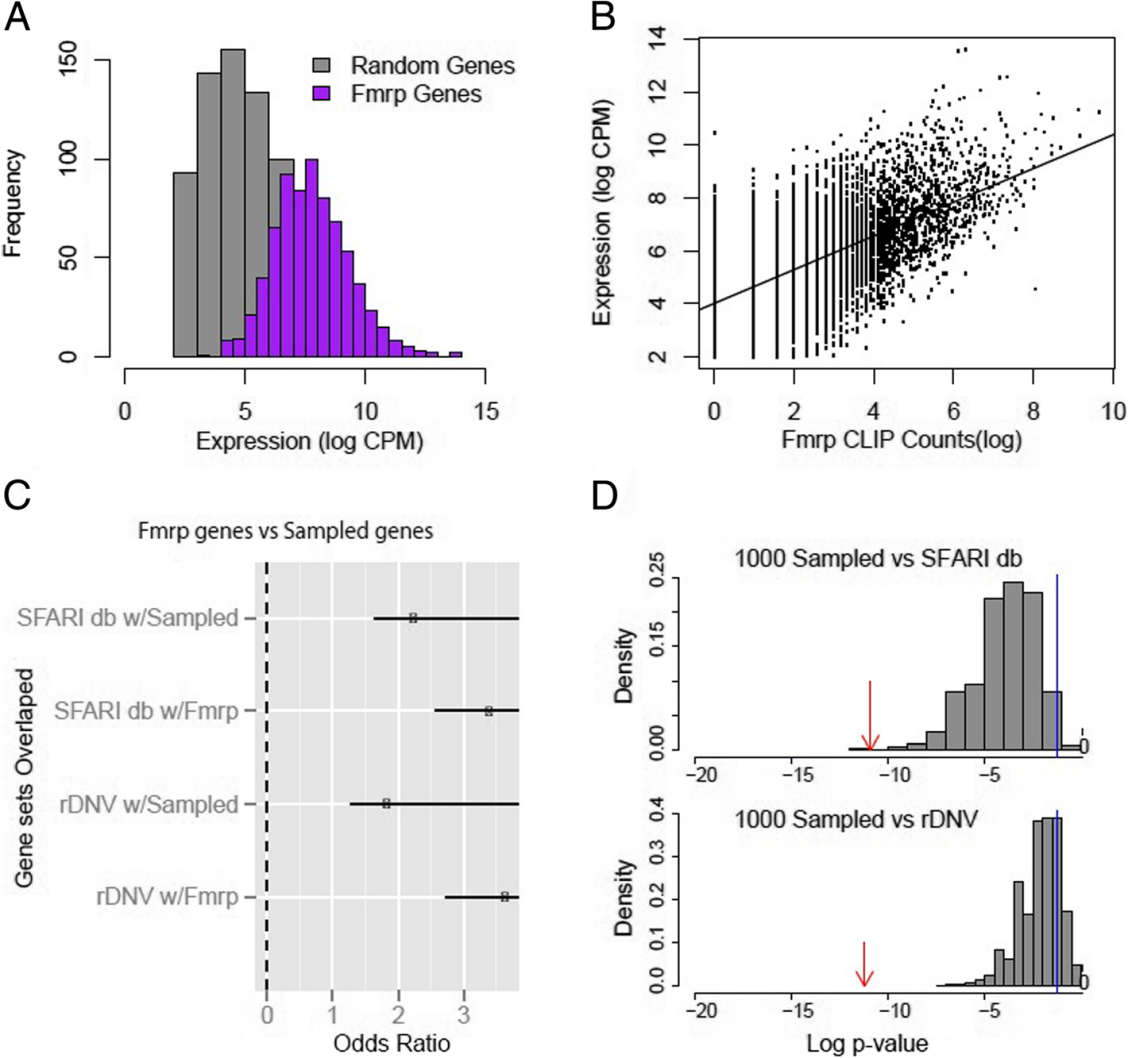
**The Fmrp targets are highly expressed in the brain, and highly brain-expressed genes overlap with autism candidate genes. (A)** Histogram of P21 mouse brain RNAseq of Fmrp targets (purple) compared to random selection brain-expressed genes (grey). Fmrp targets are significantly higher expressed, longer than random, brain-expressed genes (*P* < 2.2e − 16, *t* test). **(B)** Gene expression levels correlate moderately with Fmrp HITS-CLIP counts (0.64). **(C)** Forest plot of the odds ratio and 95% confidence intervals for Fisher’s exact test results. The Fmrp targets significantly overlap with a database of autism candidate genes (SFARI db, OR = 3.4, *P* < 1.2e − 11), recently characterized rare *de novo* variant genes in autism (rDNV, OR = 3.65, *P* < 6.1e − 12). Likewise, a sample of random genes selected to match the expression of the Fmrp targets also significantly overlap with the rDNV and SFARIdb (OR = 1.8-2.2) **(D)** Histogram of the *P* values resulting from 1,000 Fisher’s exact tests using random gene sets, each sampled to match the Fmrp target’s expression levels, compared to SFARIdb (top panel) or rDNV genes (bottom panel). Randomly sampled sets were generally less significant than the true Fmrp target list (red arrow, *P* < 1.2e − 11, *P* < 6.1e − 12), but most were more than nominally significant (blue line, *P* < 0.05).

**Figure 2:**
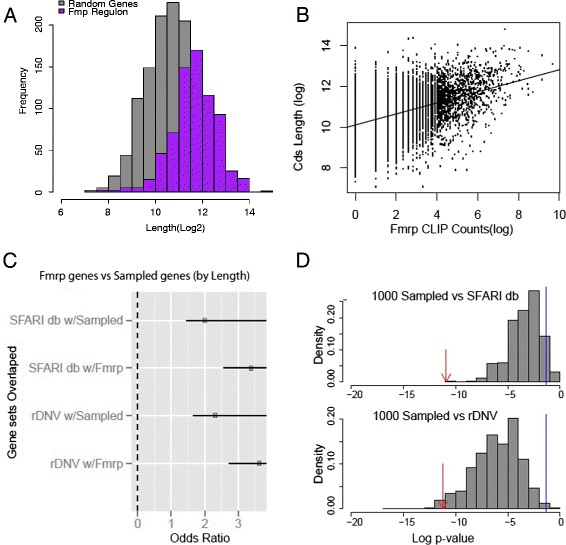
**The Fmrp targets are long transcripts, and long transcripts overlap with autism candidate genes. (A)** Histogram of coding sequence (Cds) lengths of Fmrp targets (purple) compared to an equal length list of random brain-expressed genes (grey). Fmrp targets are significantly longer than random brain-expressed genes (*P* < 2.2e − 16, *t* test). **(B)** Cds lengths correlate moderately with Fmrp HITS-CLIP counts (0.48). **(C)** A sample of random genes selected to match the expression of the Fmrp targets also significantly overlap with the rDNV and SFARIdb. **(D)** Histogram of the *P* values resulting from 1,000 Fisher’s exact tests using random gene sets, each sampled to match the Fmrp target’s Cds length, compared to SFARIdb (top panel) or rDNV genes (bottom panel). Sampled sets were generally less significant than the true Fmrp list (red arrow, *P* < 1.2e − 11, *P* < 6.1e − 12), but most were more than nominally significantly (blue line, *P* < 0.05).

There are two key facets of the reported Fmrp targets that motivated this analysis. First, as highlighted in the 2011 work [1], the Fmrp protein binds mRNA rather promiscuously, not being strongly restricted to RNAs with one particular motif in the CNS. Second, Fmrp itself is highly expressed throughout the nervous system, particularly, though not uniquely, in neurons [2]. Thus, as the confidence of Fmrp binding was dependent on read-depth, and the protein is both fairly promiscuous in sequence specificity and ubiquitously expressed in the brain, the most readily detected transcripts might be those that present the most opportunity for binding - those with the longest coding sequence and the highest expression. Thus, we tested the hypotheses that the reported Fmrp targets disproportionately represent the most abundant mRNAs in the brain (Figure 1A) and those with the longest coding sequence (Figure 2A). In support of this, using a list of genes with the highest expression in the human brain [3], one can demonstrate that the reported Fmrp targets disproportionately overlap with the most highly expressed neural genes in humans (*P* < 3e^−16^, Fisher’s exact test).

To test whether this intersection might sometimes lead to a statistical overlap between Fmrp targets and trait-associated genes in the brain, we conducted a simple experiment examining the overlap between a set of genes involved in a neurogenetic trait unrelated to Fragile X Syndrome or psychiatric disorder. It has been recognized that, since body weight tracks with consumptive behaviors, obesity is strongly influenced by genes that are expressed in the brain [4]. Thus, we tested the statistical overlap between reported genes for “obesity-related traits” and the reported Fmrp targets and see a statistical enrichment of a magnitude not too different from that sometimes seen in the literature for psychiatric disorder (*P* < 0.005,). Thus, the results of our experiment are consistent with the explanation that genes mediating any neurogenetic trait may show overlap with the reported Fmrp targets simply because both sets overlap with the set of genes highly expressed in neural tissue. Indeed, genes reported for several other neurocognitive traits also overlap Fmrp targets with nominal (*P* < 0.05) significance, for example, “hippocampal atrophy” (*P* < 0.009), “Alzheimer’s disease” (*P* < 0.022), and “cognitive performance” (*P* < 0.012).

Darnell et al. suspected a bias towards highly expressed or longer transcripts in their analysis and reported a very modest correlation between transcript abundance (cor = 0.1) and number of cross-linking and immunoprecipitation (CLIP)-seq tags ([1]; Additional file 1: Figure S1). However, their measures of abundance were based on microarray signal, which can be strongly biased by features unrelated to RNA abundance (for example, probe GC content), and lack the dynamic range of RNAseq analysis. Analyzing instead with RNAseq data from postnatal day 21, mouse cortex shows a correlation of 0.64 (Figure 1B) with expression and 0.45 with coding sequence (Cds) length (Figure 2B). Indeed, we found that a linear regression model incorporating expression, Cds length, and transcript length could account for >60% of the variance (*r*^2^) in CLIP tag number (Table 1). We would emphasize that the presence of detectable, albeit weak, motifs in the targets [5] indicates they are non-random and thus the initial findings were not exclusively driven by these features. The more likely explanation was that the sensitivity was limited to the highly expressed Fmrp targets and that many less abundant (but perhaps equally high affinity) Fmrp targets might simply have been below the threshold of detection. In the future, the comprehensive identification of Fmrp targets in the brain could be revisited with approaches to allow greater sensitivity and perhaps cell-specific normalization for transcript abundance. However, in the meantime, we tested two candidate gene lists, the Simons Foundation Autism Research Initiative (SFARI) database of curated autism candidates (SFARIdb) and the recently identified rare *de novo* variants in autism probands (rDNV) [6], to see if a statistically significant overlap with Fmrp targets could be reproduced just using equally sized sets of random genes sampled to match as best possible the transcript abundance (Additional file 1: Figure S1) or coding sequence length (Additional file 2: Figure S2) of the Fmrp target genes.

**Table 1:** **A linear model based on transcript expression and length predicts a substantial proportion of Fmrp HITS-CLIP data**

**Linear model**	**Fmrp count >1**	**Fmrp count >16**	
	***n*** **= 7,207 genes**	***n*** **= 1,228 genes**	
	***r*** ^**2**^	***r*** ^**2**^	***p***
Fmrp count ~ transcript abundance	0.41	0.21	*p* < 2.2e − 16
Fmrp count ~ Cds length	0.21	0.12	*p* < 2.2e − 16
Fmrp count ~ transcript length	0.23	0.09	*p* < 2.2e − 16
Fmrp count ~ abundance + length (either)	0.54	0.44	*p* < 2.2e − 16
Fmrp count ~ abundance + Cds length + transcript length	0.61	0.44	*p* < 2.2e − 16

Using either all genes with at least one CLIP read, expression (logCPM) or length (also log2) predicts some of the Fmrp CLIP tag depth (left column). A linear model incorporating all three has an *r* ^2^ > 0.6. Limiting the analysis only to those genes with high read count (>16), the model still has an *r* ^2^ > 0.4. All models are highly significant (*P* < 2.2e − 16).

We found in either case that random samples of abundant transcripts (Figure 1C) or transcripts with long Cds (Figure 2C) were significantly overlapped with the SFARIdb and rDNV genes, though not to the extent of that of the Fmrp targets. To make sure our results were not particular to a single sampling, we sampled 1,000 such gene lists. Most overlapped significantly with the autism candidates (Figures 1D and 2D), though again not as significantly as the reported Fmrp targets. Very similar results can be seen by comparing a contingency table overlapping the Fmrp targets and the rDNV genes relative to all brain-expressed genes (*P* < 6.02e − 12) or instead calculating the contingency table for just the genes with expression in the top quantile (*P* < 0.0002): gene expression level alone accounts for some of the overlap between rDNV genes and Fmrp targets, but not all of it.

**Figure 3:**
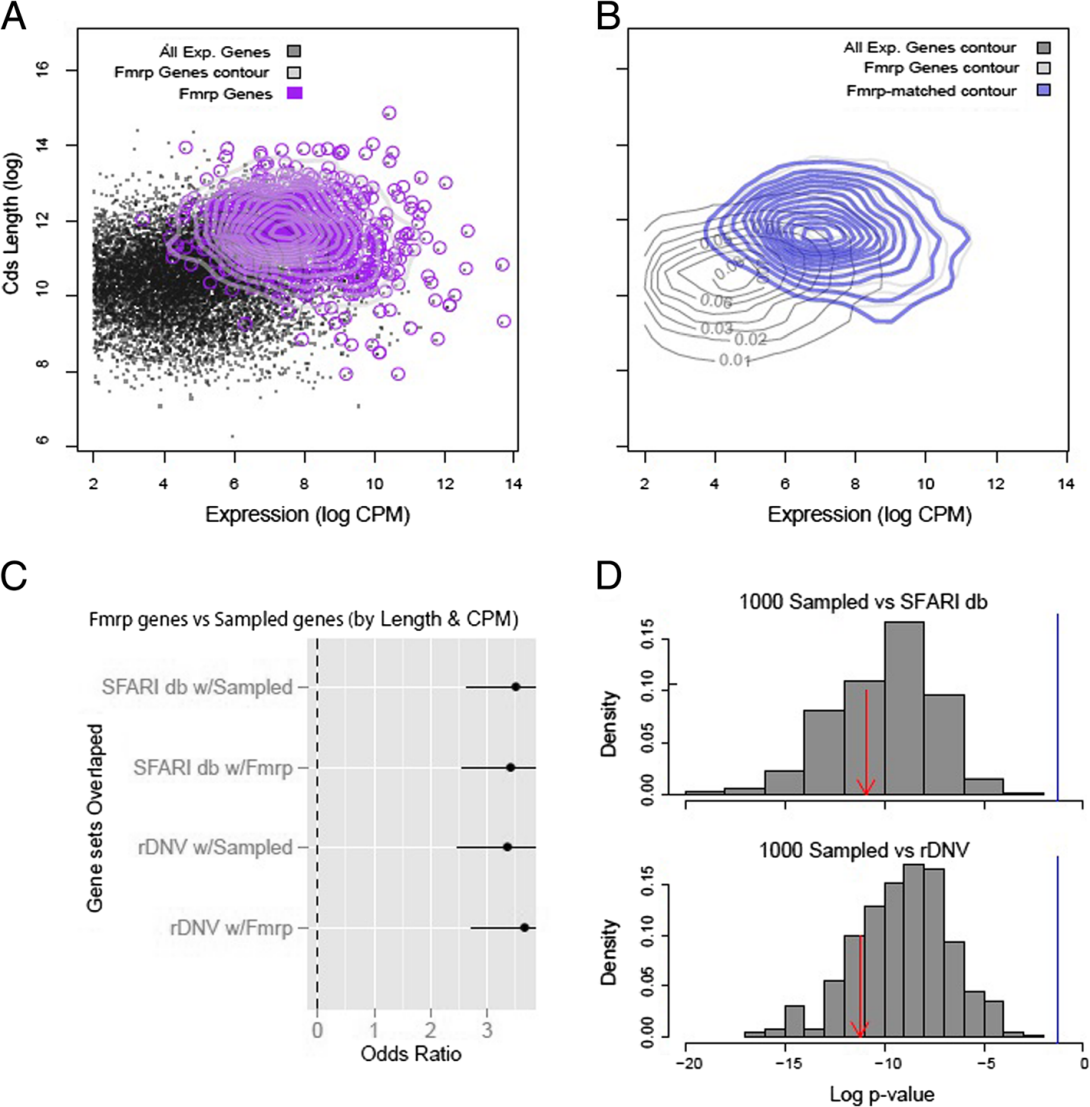
**Sets of random genes sampled to match both the length and expression overlap autism candidate genes. (A)** Scatterplot of all genes (grey dots) and Fmrp target genes (purple circles) and corresponding contours. **(B)** Contours of all genes, dark gray, Fmrp target genes (light gray), and a set of genes sampled to match the Fmrp genes (blue). **(C)** Lists of genes sampled on length and expression overlap with autism risk genes approximately as well as Fmrp target genes. **(D)** Histogram of the *P* values resulting from 1,000 Fisher’ exact tests using random gene sets, each sampled to match the Fmrp target’s both on length and expression levels, compared to SFARIdb (top panel) or rDNV genes (bottom panel). Sampled gene sets were often as significant as the true Fmrp target list (red arrow, *P* < 1.2e − 11, *P* < 6.1e − 12).

However, when instead sampling random gene sets to match the Fmrp targets simultaneously on Cds length and expression level (Figure 3), we found that a substantial fraction of the sampled lists showed an equivalent or greater statistical overlap than the original target Fmrp list (Figure 3C). Finally, we repeated these analyses but excluded the Fmrp genes from the sampling pool. Because the Fmrp genes so strongly monopolize the long and highly expressed gene space (Figure 3A), it was impossible to sample a set of genes that perfectly matched the two-dimensional distribution of the Fmrp targets after excluding those genes (Additional file 3: Figure S3A, B). Nonetheless, a random set of non-Fmrp target genes, or simply a list of the longest and highest expressed non-Fmrp target genes, also significantly overlapped both of the autism candidate lists (Additional file 3: Figure S3C). This demonstrates that long, highly expressed genes in the brain tend to overlap with disease-risk genes, whether they are on the Fmrp target list or not. It is worth noting that a disproportionate amount of long and highly brain-expressed genes is not just a feature of the Fmrp targets, but of course many other sets of genes important for the functioning of the nervous system (for example, Gene Ontologies terms for ‘Synapse’ or proteomics studies of synaptic proteins), so some of the conclusions here extend beyond consideration of just the Fmrp targets. Likewise, genes with enriched expression in the brain are on average longer than genes expressed in other tissues [7], and thus any analysis that identifies long genes may tend to overlap statistically with brain-expressed genes, Fmrp targets, autism candidates, synaptic proteins, etc.

## Conclusions

We have shown that the overlap between the reported Fmrp targets and at least two autism candidate gene lists can be reproduced by simply selecting for similarly long and highly expressed genes in the brain. This is consistent with long and highly brain-expressed genes also being more likely to be under selective constraint [8] or containing critical exons [9] and provides a straightforward explanation for why the Fmrp target list overlaps so frequently with sets of genes implicated in psychiatric disease by genetic studies. It is imaginable that similar features may explain why the Fmrp target list also frequently overlaps results of brain transcriptomic studies as well. This parsimonious explanation thus obviates complex hypotheses which require the Fmrp protein itself to be involved in the mechanism for many diverse disorders or different forms of ASD. Of course, mutations in Fmrp clearly do still cause Fragile X Syndrome, the most common form of monogenic ASD, and thus continued research into this protein remains important for that reason alone.

This model also provides reasonable explanations for two other puzzles about the Fmrp targets. First, it could explain why studies of the Fmrp targets in HEK cells [10] are less concordant with other studies [5] and why HEK cell data overlaps marginally if at all with psychiatric disorder candidate gene lists [11]. The HEK cell data should be biased towards long, highly expressed genes in HEK cells, which will likely contain few neural-specific transcripts. Second, this model might explain why identifying strong *cis* motifs or other features in the RNA that might mediate Fmrp binding has proven challenging [5]. Efforts to model the affinity of Fmrp for particular mRNAs will likely be aided by first removing the variance in the Fmrp CLIP data that can be explained by transcript length, Cds length, and transcript abundance. The authors of [1] suspected a bias towards highly expressed genes, but recognized the data were not available at that time to adjust for it, particularly if the level of Fmrp varies substantially across cell types in the brain. Thus, the definition of the Fmrp targets can probably now be revisited both with greater sensitivity and by models incorporating these covarying factors to identify additional features of the transcripts that account for the remaining variance in Fmrp binding.

In the end, it may well be that these studies find that Fmrp does bind preferentially to those transcripts whose protein levels most require precise regulation for normal CNS function. It is not unreasonable that this set of genes would also be vulnerable to haploinsufficiency [8,9] and of course be expressed highly in the brain. And a set of genes needing more precise regulation may indeed be selected by evolution to be longer (that is, allowing more potential sites for regulatory motifs). Thus, Fmrp binding may have been serving as a useful proxy for these other features. However, in the interim, we have provided a table (Additional file 4: Table S1) with precalculated weightings for length, expression, or length and expression for measurably brain-expressed genes. This can be used for drawing random samples for comparison to candidate gene lists, to help determine whether the candidate list is enriched in Fmrp targets specifically and/or long, highly brain-expressed transcripts generally.

## Methods

### Comparisons to GWAS and GTEX

Eight hundred forty-two Fmrp targets were identified from Supplemental Table 2 of [1]. Genes associated from cognitive traits were downloaded from the NHGRI GWAS Catalog [12]. Highly expressed genes in the brain were defined as the 842 genes with the highest average RPKM across all brain samples in the genotype-tissue expression (GTEX) collection [3] (1/31/13 data release, summarized to genes, all brain samples averaged). Statistical overlap was calculated in *R* using the Fisher’s exact test, right-side probability, genome size of 20,000.

### RNAseq

All experiments involving mice were approved by the Washington University Animal Studies Committee. For each replicate, cortical dissections were performed on three C57BL/6 male mice 21 days post birth. Tissue was homogenized in standard homogenization buffer (10 μL/mL pH 7.5 tris-Cl (Invitrogen 15567–027), containing 0.25 M sucrose (IBI IB37160), 1 μl/mL RNasin (Promega N251B), SuperRNasin (Ambion AM2696), protease inhibitor cocktail Tablet 1 per 10 mL (Roche 04693132001), 1 mM tetrodotoxin citrate (Tocris Bioscience 1069), and 0.5 mM DL-dithiothreitol (646563-10X) of which was centrifuged for 10 min at 1,000 rcf. The supernatant was then treated with the addition of 10X lysis buffer (10% IGEPAL (Sigma I8896-50ML), 300 mM DHPC (Avanti 850306P), 100 mM HEPES (Sigma H0887), 1.5 M KCl (Ambion AM9640G), and 50 mM MgCl2 (Ambion AM9530G)) for 10 min and centrifuged again for 15 min at 20,000 rcf. RNA was collected from 60 μL of supernatant on QIAGEN RNeasy MINI Kit (74106) with 2-mercaptoethanol (Sigma M7522) and DNase treatments (Qiagen 79254). Sequencing libraries were amplified (21 cycles) using Nugen Amplification Kit Ovation® RNA-Seq System V2 (7102). Standard Illumina adapter ligation, library preparation, and sequencing were performed on an Illumina Hi-seq by the Genome Technology Access Center at Washington University in St. Louis. Resulting reads were trimmed for quality and contaminating adapters. Possible rRNA contamination was filtered out by aligning with Bowtie2 to rRNA sequences from GenBank, ENSEMBL, and UCSC’s RepeatMasker track. Remaining sequences were then mapped to the Ensembl 75 mouse genome. Counts per million reads (CPM) for each gene were quantified using HTSeq. Data represent the average of three biological replicates.

### Comparisons to length and expression

We then intersected this data with Supplemental Table 2C of Darnell et al. for all genes with a matching gene symbol and extracted Fmrp high-throughput sequencing of RNAs isolated by cross-linking immunoprecipitation (HITS-CLIP) tag count (Fmrp.sum), Cds length, and transcript length. For sampling analyses, we used all genes with measurable expression in the brain (logCPM > 2 in RNAseq data), as only brain-expressed genes could have been captured by a brain HITS-CLIP experiment and further filtered to keep only those genes with an annotated Cds and transcript lengths (final effective genome size = 9,544). All variables were converted to Log2 scale for normality prior to correlation and linear regression, and for these analyses genes with <1 CLIP tag were excluded (final gene number, 7207). A spreadsheet aggregating all of these variables is provided (Additional file 4: Table S1).

### Candidate gene lists

For the SFARIdb analysis, we used the list of all unique genes (gene-score table, as downloaded on 8/7/14); rDNV genes are from Supplemental Table seven from [6], the dnv_LGDs_prb column.

### Sampling random gene sets

To generate sets of 716 random genes with the same distribution of expression as the 716 Fmrp genes surviving the filters above, we computed a kernel density estimate on the logCPM (function ‘density’ in R) as well as a kernel density estimate on all 9,544 genes and used the ratio of these to assign probabilities for sampling to all 9,544 genes in the genome based on their expression levels. A similar sampling was done based using a kernel estimated from the Cds length or a 2d kernel (function kde2d) on both length and expression. Fisher’s tests were calculated as above for overlap between sampled lists with a genome size of 9,544. For !Fmrp lists, sampling was conducted on the 8,828 non-Fmrp genes, but with the same probabilities as above, or taking those of the 8,828 with the highest probabilities (Top !Fmrp). All sampling is without replacement.

## Additional files

Additional file 1: Figure S1. Sampled distributions for expression approximately match the distribution of Fmrp genes. The distribution of the Fmrp target genes (purple) is markedly higher than a set of brain-expressed genes drawn at random from the genome (grey). Our sampling using our weightings (blue) can approximate the distribution of the Fmrp genes. Additional file 2: Figure S2. Sampled distributions for Cds length approximately match the distribution of Fmrp genes. The distribution of the Fmrp target genes (purple) is markedly higher than a set of brain-expressed genes drawn at random from the genome (grey). Our sampling using our weightings (blue) can approximate the distribution of the Fmrp genes. Additional file 3: Figure S3. Transcripts sampled jointly for length and expression cannot completely approximate the Fmrp genes. The 2d distribution density of the Fmrp target genes (light gray) is markedly shifted compared to brain-expressed genes in general (dark grey) and random samples of genes when Fmrp targets are excluded (red, A). Even taking those with the top remaining probabilities (pink, B), can’t perfectly match the length and expression of the Fmrp target genes. However, (C) gene lists sampled after excluding the Fmrp target genes also significantly overlap with the SFARIdb and rDNV genes. Additional file 4: Table S1
**Data.** Columns 1 to 5 from Darnell et al., Table S2C, columns of same names. Total length, Cds length, and Fmrp.sum have been log2 transformed. The Fmrp targets were those with an FDR < 0.01. Columns 6 to 9 from P21 cortical RNAseq data. Columns 10 to 12 are weightings for each gene by length, expression, or length and expression to allow sampling for random gene lists that match as best possible these features of the Fmrp targets.

## Abbreviations

Fmrp: fragile X mental retardation protein
GTEX: genotype-tissue expression
CLIP: cross-linking and immunoprecipitation
SFARI: Simons Foundation Autism Research Initiative
CPM: counts per million reads
Cds: coding sequence
GWAS: genome-wide association study
