# Supplementary figures and images for: Fmrp targets or not: long, highly brain-expressed genes tend to be implicated in autism and brain disorders
Source: Mol Autism. 2015 Mar 11;6:16. doi: 10.1186/s13229-015-0008-1 (PMC4363463; doi:10.1186/s13229-015-0008-1)

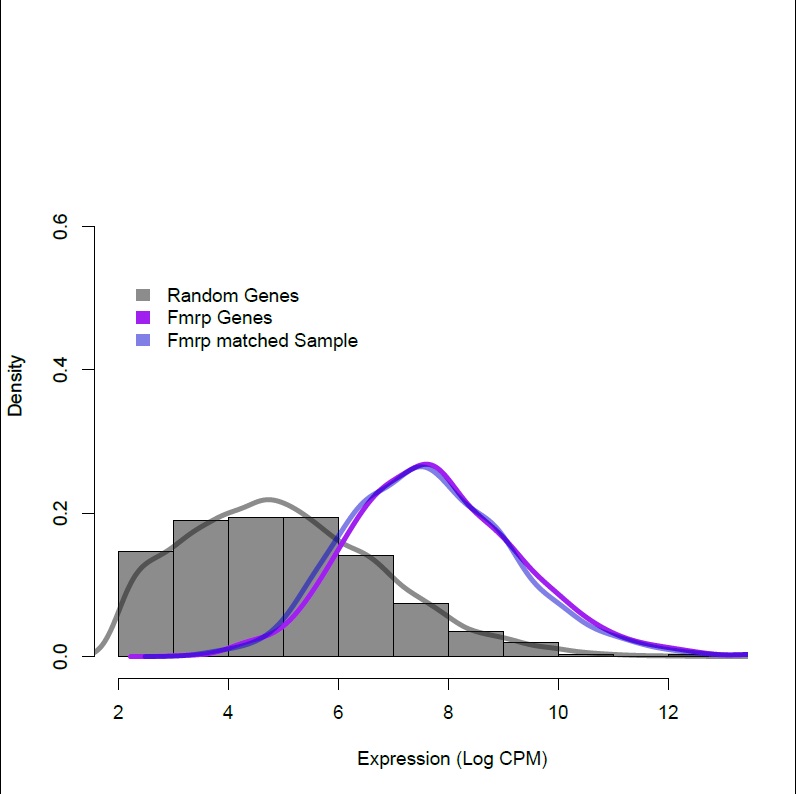

Supplement: Additional file 1: Figure S1. — Sampled distributions for expression approximately match the distribution of Fmrp genes. The distribution of the Fmrp target genes (purple) is markedly higher than a set of brain-expressed genes drawn at random from the genome (grey). Our sampling using our weightings (blue) can approximate the distribution of the Fmrp genes. [file 13229_2015_8_MOESM1_ESM.jpeg]

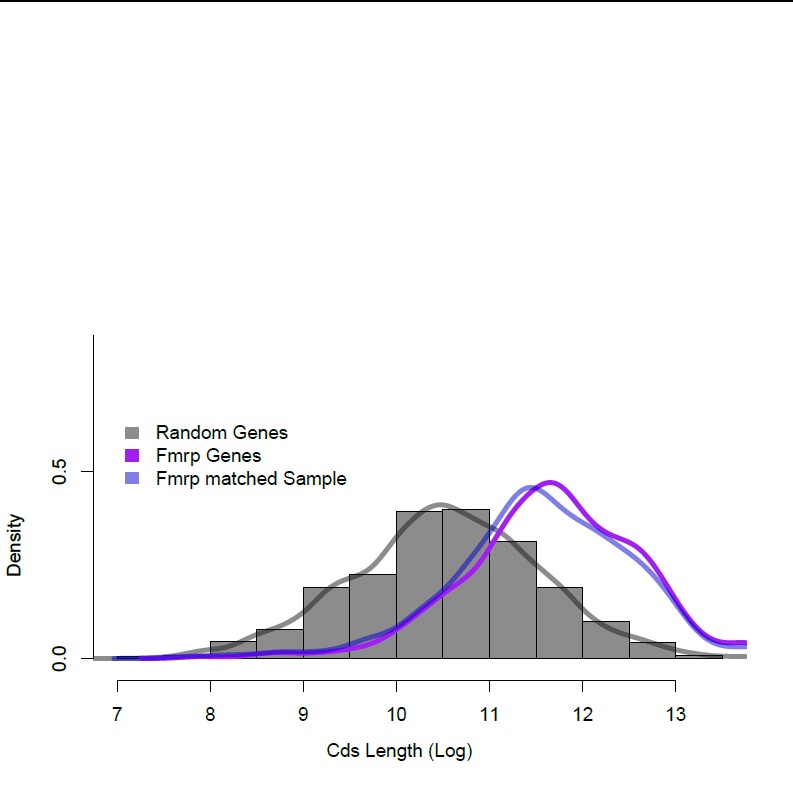

Supplement: Additional file 2: Figure S2. — Sampled distributions for Cds length approximately match the distribution of Fmrp genes. The distribution of the Fmrp target genes (purple) is markedly higher than a set of brain-expressed genes drawn at random from the genome (grey). Our sampling using our weightings (blue) can approximate the distribution of the Fmrp genes. [file 13229_2015_8_MOESM2_ESM.jpeg]

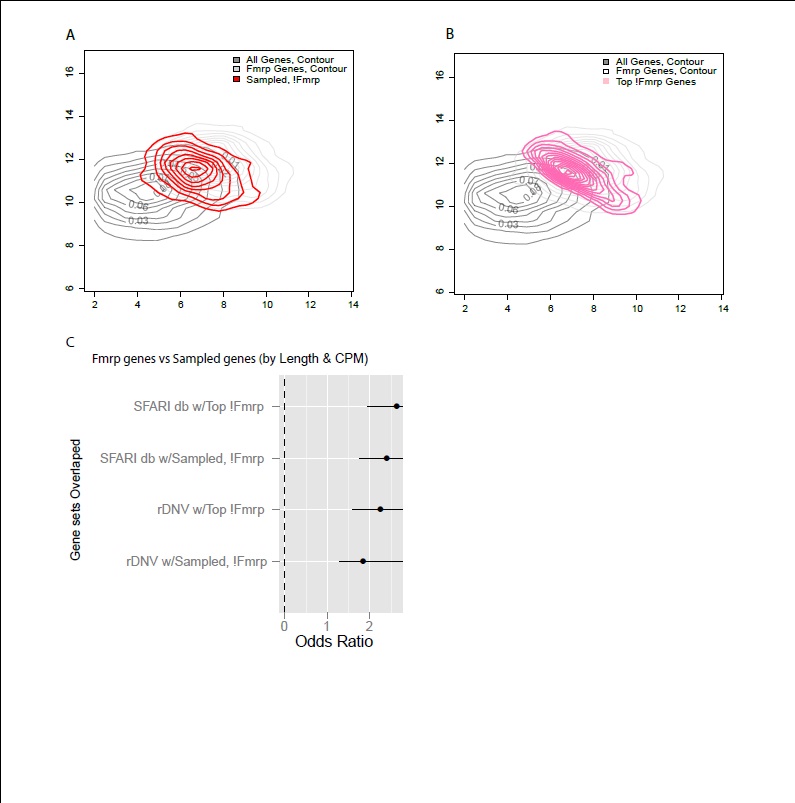

Supplement: Additional file 3: Figure S3. — Transcripts sampled jointly for length and expression cannot completely approximate the Fmrp genes. The 2d distribution density of the Fmrp target genes (light gray) is markedly shifted compared to brain-expressed genes in general (dark grey) and random samples of genes when Fmrp targets are excluded (red, A). Even taking those with the top remaining probabilities (pink, B), can’t perfectly match the length and expression of the Fmrp target genes. However, (C) gene lists sampled after excluding the Fmrp target genes also significantly overlap with the SFARIdb and rDNV genes. [file 13229_2015_8_MOESM3_ESM.jpeg]
